# Clinical Insights into Antipsychotics and Rifampicin Interaction: A Case Report

**DOI:** 10.1192/j.eurpsy.2024.1456

**Published:** 2024-08-27

**Authors:** P. Abreu, C. Ferreira dos Santos, M. Pereira, M. Vieira Lisboa, M. Cameira, M. Rebelo Soares

**Affiliations:** Centro Hospitalar Psiquiátrico de Lisboa, Lisbon, Portugal

## Abstract

**Introduction:**

Antipsychotics are the primary class of drugs used to manage schizophrenia. These medications help control and reduce the severity of these symptoms, allowing individuals with schizophrenia to better function. On the other hand, rifampicin, used as treatment for tuberculosis, is a powerful inducer of several drug-metabolizing enzymes which have the potential to decrease the plasma levels of antipsychotics. Therefore, the presence of multiple pharmacokinetic interactions can alter how antipsychotics are metabolized, leading to a notable clinical impact when these medications are administered concurrently.

**Objectives:**

The objective is to share valuable clinical experiences and insights to aid healthcare providers in making informed decisions when faced with the challenge of co-administering antipsychotics with rifampicin, ultimately ensuring the safety and efficacy of treatment for their patients.

**Methods:**

It will be discussed a case of a 41-year-old woman with the diagnosis of schizophrenia under treatment with paliperidone palmitate and clozapine who had a sudden relapse after starting treatment for latent tuberculosis with rifampicin as a framework for a literature review based off Pubmed.

**Results:**

The antituberculosis drug rifampicin induces drug-metabolizing enzymes in the liver, having the greatest effects on the expression of cytochrome P450 (CYP3A4) and therefore can lead to a decrease in the plasma levels of antipsychotic medications that also rely on these pathways for clearance. In this particular case, although specific data on clozapine and paliperidone concentrations were not reported, fluctuations in symptomatology following rifampicin introduction were probably explained by an inducing effect of this drug on their metabolism. So, when initiating rifampicin treatment and when discontinuing it, clinicians should carefully assess the dosages of any concomitant medications that may potentially interact with rifampicin. To ensure effective therapy during rifampicin treatment, it is crucial to monitor both the patient’s clinical response and their blood drug concentrations, making dosage adjustments as necessary.

**Conclusions:**

This case report offers valuable guidance to clinicians on safely and effectively managing drug interactions between antipsychotic medications and rifampicin, ensuring the well-being of their patients during treatment. The co-administration of these medications lacks robust clinical evidence, and notably, there is insufficient data regarding its impact on plasma antipsychotic levels, a crucial factor in determining clinical effectiveness.

**Disclosure of Interest:**

None Declared

